# Transcriptome Analysis of Short Fiber Mutant Ligon lintless-1 (Li_1_) Reveals Critical Genes and Key Pathways in Cotton Fiber Elongation and Leaf Development

**DOI:** 10.1371/journal.pone.0143503

**Published:** 2015-11-24

**Authors:** Wenhua Liang, Lei Fang, Dan Xiang, Yan Hu, Hao Feng, Lijing Chang, Tianzhen Zhang

**Affiliations:** State Key Laboratory of Crop Genetics and Germplasm Enhancement, Cotton Hybrid R & D Engineering Center, the Ministry of Education, Nanjing Agricultural University, Nanjing, 210095, China; East Carolina University, UNITED STATES

## Abstract

For efficient spinning and superior fabric production, long fiber length is a desired trait for cotton production. To unveil the molecular basis of the cotton fiber length regulation, a short fiber mutant, Ligon lintless-1 (Li_1_), is selected to compare with its corresponding wild type (WT). Li_1_ is a monogenic dominant cotton mutant causing extremely short fibers (<6mm) on mature seeds with visible pleiotropic effects on vegetative growth and development. In this research, we compared the transcriptome of fiber bearing ovules at 1 DPA, 3 DPA, 8 DPA and leaf between Li_1_ mutant and WT. A total of 7,852 differentially expressed genes (DEGs) were detected in ovules and leaves, which mainly participated in sugar, secondary metabolite and lipid metabolism pathways based on KEGG analysis. The common DEGs at 1 DPA and 3 DPA were involved in the responses to endogenous stimulus, signal transduction and long-chain fatty acid biosynthesis. For 3 DPA, 8 DPA and leaf, the common DEGs were involved in the responses to auxin and receptor kinases related pathway. Further analysis showed that 37 genes involved in very-long-chain fatty acid biosynthesis were suppressed in Li_1_ mutant during fiber fast elongation development. Most of the DEGs involved in cell wall metabolism, such cellulose synthase, expansin family, and glycosyl hydrolase were differentially expressed at 3 DPA and 8 DPA. Our results provide new insights into the mechanisms of fiber elongation, and offer novel genes as potential objects for fiber length improvement.

## Introduction

Cotton fibers are highly elongated single-celled seed trichomes that emerge from the outer epidermal cells of ovules on or around the day of anthesis (DOA). Only about 25%-30% of the ovule epidermal cells differentiate into spinnable lint fiber cells. The development of the fibers includes four well-defined stages [1–3]. The first stage is the initial development period, which lasts from 3 days before anthesis to 3 days post-anthesis (DPA). Following the initiation stage, cotton fibers enter the second developmental stage, known as the period of fiber elongation. Fiber elongation continues to nearly 22 DPA, with peaks at approximately 6 to 12 DPA [2–5]. During this stage, fiber cells can grow at rates of more than 2 mm/day [6–8]. The secondary cell wall (SCW) biosynthesis stage is the third stage of fiber development, which usually begins at 12–16 DPA in field conditions [6, 7, 9, 10], and this process persists until the fiber maturation stage. When bolls crack and open at 40–60 DPA, fibers desiccate and mature. Fiber properties are largely quantitative traits and are influenced by environmental and genetic factors [7, 11–13].

Cotton fiber mutants are a powerful resource for the elucidation of fiber development mechanisms, owing to the morphological and biochemical variances in their fiber cells. In cotton there are two mutant lines, Ligon Lintless-1 and Ligon Lintless-2 (Li_1_ and Li_2_) [14, 15], that exhibit extremely short lint fibers of approximately 6 mm on mature seeds. The two fiber mutations are monogenic dominant mutations characterized by short fibers [15, 16]. Cytological studies showed no differences in the appearance of seed fibers in Li_2_ mutants compared to wild types (WT) during the initiation and early elongation stages, suggesting that the effects of the mutation most likely occur during the fiber elongation stage [17–18]. Although Li_1_ mutant seed fibers undergo initiation and elongation in a similar manner to WT fibers from 0 to 2 DPA, Li_1_ fibers begin to show slightly distorted morphological features at 3 DPA; suggesting that their gene expression changes as early as during or possibly even before this stage [19]. Hence, the two seed fiber mutants, Li_1_ and Li_2_, form an outstanding system for studying the molecular mechanisms of fiber elongation.

In a previous study, an expressed sequence tag simple sequence repeat (EST-SSR) marker with complete linkage to the Li_2_ genetic locus on chromosome 18 was identified, while the Li_1_ gene was mapped to chromosome 22 with SSR [19] and RFLP [20] analyses. Recently, Gilbert et al. [21] analyzed 2,553 F_2_ progeny using SSR markers for fine mapping of the *Li*_*1*_ gene. Their results showed that the *Li*_*1*_ gene was mapped between two markers, TMB2500 and DPL0489, which had a genetic distance of 0.08cM and 11.6cM from the *Li*_*1*_ gene, respectively. A total of 23 genes were found to reside between the two markers [21]. Next generation sequencing offers new ways to identify the genetic mechanisms that underlie mutant phenotypes. Recently, Thyssen et al. [22] used multiple high throughput sequencing techniques to predict novel SNP markers for fine mapping the *Li*_*2*_ gene. Between the two SNPs of CFB 5851 and CFB 5852, only one gene, an aquaporin, has been identified, and it is highly expressed in WT fibers. This may be an important candidate gene for *Li*_*2*_ traits, based on the current evidence [22].

Unlike the Li_2_ mutant, the Li_1_ mutant appears a to have a pleiotropic phenotype in the form of abnormal development with regard to both fiber tissues and vegetative tissues, such as controlled leaves, stems, and flowers. The Li_1_ mutant shows a distorted phenotype that is visible at the seedling stage [23], and Li_1_ seedlings also appear to have a lower survival rate than the other cotton. The short fibers of Li_1_ mutants have been used to study the developmental processes of both primary and secondary cell walls [24–27]. Previous microarray studies identified over 100 differentially expressed transcripts in 24 DPA fibers, including SuSy, Expansins, and Myb transcription [24]. The proteome profiles of 6 DPA fibers revealed no obvious difference in protein expression, while 81 proteins were differentially expressed between mutant and WT fibers at 12 DPA [27]. Recently, a microarray experiment analyzed the fiber initiation and elongation stages of 0 DPA ovules, and 3 DPA and 6 DPA fibers from Li_1_ mutants, and found that auxin signaling, sugar signaling, gibberellins, and ethylene-related pathways are active in the early stages of fiber elongation [26]. Another recent gene expression profiling study of fiber development at 3 DPA, 12 DPA, and 16 DPA indicated some of the key hormone-related metabolic pathways were severely repressed during elongation and secondary cell wall synthesis [21]. In addition, transcriptome sequencing of leaf tissues showed that transcription factors related to leaf morphogenesis are significantly differentially expressed between the Li_1_ mutant and WT. Several fiber development-related genes were also found to be down regulated in the mutant leaf transcriptome, such as heat shock protein family genes, cell wall synthesis-related genes, energy metabolism-related genes, and WRKY transcription factors. Pathway analysis revealed that a number of pathways related to fiber elongation were down regulated at different fiber development stages [28]. Analysis of multiple transcriptome profiles obtained from Li_1_ and Li_2_ mutants and their WT showed that the expression patterns of eighty-eight differentially expressed genes (DEGs) were altered in both short fiber mutants under different growth conditions [29].

Despite years of research, the mechanisms behind the effect of the Li_1_ mutation on the transcript profile at the fiber early elongation and leaf morphogenesis are still unclear. Previous studies have mainly used microarray experiments, and the number of probes is limited, thus, affecting the detection of DEGs. Moreover, microarrays are unable to detect unknown transcripts. In this study, we report the first use of the newly published tetraploid *G*. *hirsutum* (TM-1) whole genome sequence [30] with Illumina next generation sequencing (NGS) technology to pursue RNA-seq analysis of global gene expression changes in Li_1_ mutants compared to the wild type. Furthermore, we used the tetraploid cotton genome sequence as a reference genome to allow accurate determination of the position of reads in order to effectively analyze differentially expressed genes.

## Methods

### Plant materials and growth conditions

**Fig 1:**
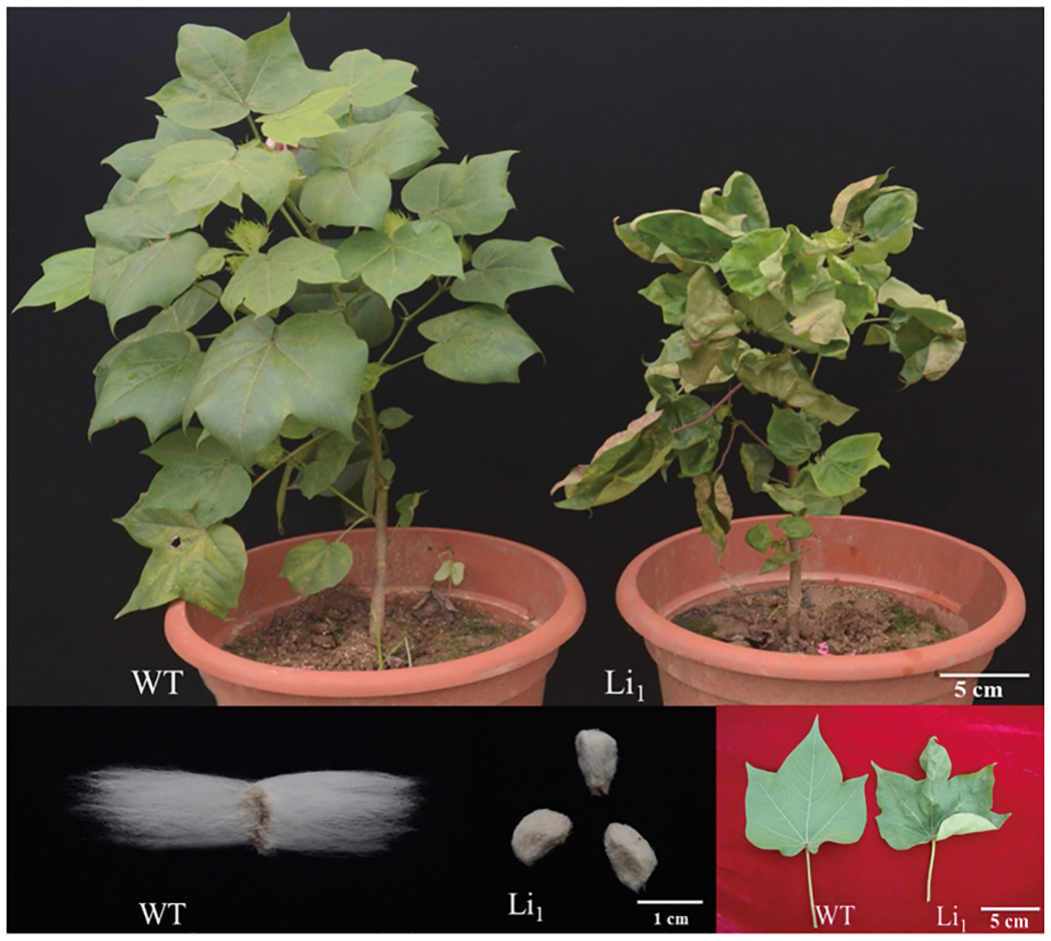
Comparison of the phenotypes of WT and Li_1_ mutant seed fibers and leaves.

The Li_1_ mutant and its corresponding WT were derived from plants that had been self-pollinated for at least 5 generations in our laboratory (Fig 1). These plants were provided by Dr. Kohel (USDA-ARS, College Station, TX, USA) and used for transcriptome analysis. These materials were planted in the Jiangpu experimental field of Nanjing Agricultural University, Nanjing, Jiangsu Province, China. For analysis of the leaf transcriptome, we collected young leaves from at least 3 Li_1_ plants and 3 WT plants as three biological replicates, respectively. Ovule samples were harvested at -3 DPA, -1 DPA, DOA, 1 DPA, 3 DPA, 5 DPA, and 8 DPA, placed on ice immediately, and then samples of each stage were randomly grouped into 3 individual replicates in the laboratory. The samples were dissected, immediately frozen in liquid nitrogen, and stored at -70°C for subsequent experiments. Samples were collected at each developmental stage and were tagged on the same day. A small number of the samples collected at the -3 to 5 DPA time-points were used for scanning electron microscope (SEM) analysis.

### RNA Isolation and qPCR Analysis

Total RNA was extracted from Li_1_ and WT samples using the CTAB method [31]. The RNA quality and purity was determined by its RNA integrity number (RIN), which was measured using an Agilent Bioanalyzer 2100 (Agilent Technologies Inc., Santa Clara, CA, USA).

The same RNA samples were also used for real-time quantitative PCR (qPCR) analysis. Gene-specific qPCR primers were designed using SNAP Program (http://ausubellab.mgh.harvard.edu/) and synthesized commercially (Genscript, Nanjing, China). *Histone3* (AF024716) was used as an internal control. The qPCR was performed using IQ SYBR Green Supermix (Bio-Rad, USA) according to the manufacturer’s instructions in an ABI 7500 sequence detection system, as described in the manufacturer’s protocol (Applied Biosystem). A dissociation curve was generated and used to validate that a single amplicon was present for each qPCR reaction. The relative expression levels were calculated using the 2^-Δt^ method.

### Library preparation and illumina sequencing

All RNA samples were tested for quality using a spectrophotometer and an Agilent 2100 Bioanalyzer: RNA was only accepted if it was uncontaminated and the RIN was greater than 8.0. Approximately 2.0μg of total RNA was then polyA selected and chemically fragmented to about 350bp, and cDNA was created using random hexamer primers. Library preparation followed the TruSeq Illumina protocol with each individual library receiving a unique Illumina barcode, allowing for their identification after multiplexed sequencing. RNA-seq was performed on an Illumina HiSeq 2000 platform with paired-end (PE) sequencing reads (2×100 bp). The Li_1_ mutant phenotype involves both fiber tissues and vegetative tissues, such as leaves and stems. To illuminate the molecular mechanisms controlled by the *Li*_*1*_ gene that are involved in the development of stunted and deformed plants and fibers, we selected fiber bearing ovules at three stages of development (1 DPA, 3 DPA, and 8 DPA) as well as leaf tissues. Three biological replicates for leaf tissues and three stages ovules of Li_1_ mutant and WT were used to create 24 independent libraries that were sequenced using the Illumina HiSeq 2000 sequencing platform.

### Identification and functional analysis of differentially expressed genes

The raw FASTQ format data sets generated by CASAVA v1.8.2 software and FASTQC v0.10.1 [32] and FASTX toolkit v0.0.13 software [33] were used for the quality assessment of sequencing reads. Reads contaminated with Illumina adapters were detected and removed by Trimmomatic software (Released Version0.22, www.usadellab.org/cms/index/php?page=trimmomatic). Poor quality reads (Phred score <20) were trimmed from both ends with SolexaQA package v2.0 [34]; only reads with lengths ≥ 35bp on both sides of the paired-end format were subjected to further analysis. Tophat2 software (v2.0.12) was used to process the clean reads mapping to the latest release of the *Gossypium hirsutum* L. (TM-1) genome [30] with the following parameters: -r 130 –mate-std-dec 30—no-novel-juncs. The TM-1 genome contains 70,478 predicted protein-coding genes, and the information of these genes was showed in GFF3 file [30]. After each library was mapped to the reference genome, the annotation GFF3 file from the TM-1 genome was used as a reference for Cuffdiff (v2.2.1) software to calculate the FPKM (fragments per kilobase of transcript per million mapped reads) value of each transcript in the mutant and WT and to test the statistical significance of any differences between them [35, 36]. Only transcripts with an FPKM ≥ 1 were considered to be expressed. The differentially expressed transcripts were identified with q value ≤ 0.05 and a fold-change of ≥ 2 between the Li_1_ mutant and WT.

The DEGs between the Li_1_ mutant and WT underwent K means clustering with the open-source program Cluster 3.0 (http://bonsai.hgc.jp/~mdehoon/software/cluster/software.htm). We also performed clustering with the ‘Self-organizing tree algorithm’ (SOAT, Multiple Array View software, Mev 4.9.0) [37]. GO enrichment and Kyoto Encyclopedia of Genes and Genomes (KEGG) pathway analysis [38] were performed using the BLAST2GO database (http://www.blast2go.com). The enriched GO terms were visualized using ReviGO [39], and MapMan software was also used to analyze DEG enrichment and metabolic pathways [40].

In the present study, 1 DPA, 3 DPA, and 8 DPA ovule and leaf tissues were employed for transcriptome analysis. Fortunately, sequencing of the upland cotton genome has been completed in our laboratory [30]. This provides a useful resource for the study of tetraploid cotton transcriptomes.

## Results

### Morphology of Li_1_ fibers during initiation and early elongation

The Li_1_ mutant gene produces pleiotropic effects on multiple tissues and organs such as fibers, leaves, stems, and flowers.

**Fig 2:**
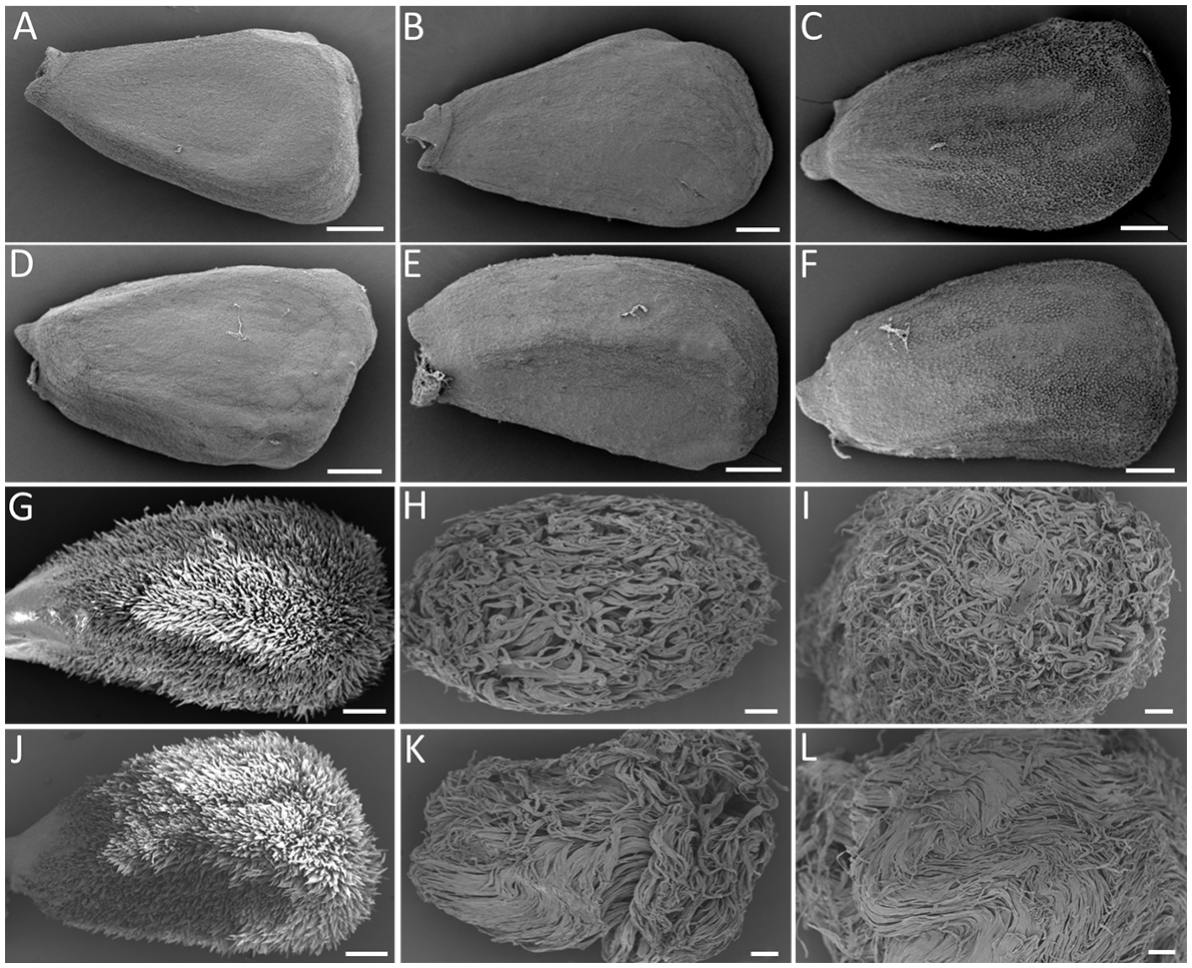
SEM analysis of developing fibers and ovules from Li_1_ mutant and WT plants. Comparison of Li_1_ (A-C, G-I) and WT (D-F, J-I) fibers and ovules prior to and during fiber initiation and early elongation. The developmental time-points shown are: (A, D) -3 DPA; (B, E) -1 DPA; (C, F) DOA; (G, J) 1 DPA; (H, K) 3 DPA; (I, L) 5 DPA. The scale bars in all panels are 200 μm.

SEM analyses showed no obvious differences in the morphology of ovules and fibers between Li_1_ mutant and WT plants from -3 DPA to 1 DPA (Fig 2). The ovule epidermal cells of mutants underwent normal initiation at 0 DPA and the morphology of ovule surfaces displayed no significant differences to the WT (Fig 2C and 2F). Likewise, the fiber morphology and length appeared to be similar at 1 DPA (Fig 2G and 2J; S1A and S1B Fig). However, significant differences in fiber elongation between mutant and WT were observed at 3 DPA. Li_1_ fiber elongation appeared to be abnormal at the surface of ovule at this stage (Fig 2H and 2K; S1C and S1D Fig), and there were some minor differences in fiber length at 5 DPA. Therefore it can be inferred that the mutant fiber phenotype showed obvious differences as early as 3 DPA. These results demonstrate that the short fiber phenotype in the Li_1_ mutant is largely due to gene regulation at early stage of fiber elongation. In view of its extraordinary pleiotropic effect on different tissues and organs of plant, the *Li*_*1*_ gene could be an important regulatory factor in key metabolic pathways, and influences the morphology of fibers and other tissues.

### RNA-Seq of ovule and leaf tissues from Li_1_ mutant and wild type plants

To assess global transcriptome changes occurring in Li_1_ mutant, total RNA samples were extracted from leaf and ovule tissues before abnormal fiber development at 1 DPA, at the critical development time point of 3 DPA, and at 8 DPA, when significant differences in fiber length are observed [26]. In total we created twenty-four independent libraries including three biological replicates that were sequenced using the Illumina HiSeq 2000 sequencing platform. A total of 381,405,732 Li_1_ and 485,095,352 WT raw paired-end reads with a length of 100 bp were obtained from ovule and leaf tissues by Illumina sequencing, and on average there were 36,104,212 raw reads from each library. The raw reads were trimmed with Illumina adapters and low quality bases were filtered out using various techniques. In total 347,931,468 and 447,297,594 clean reads were obtained from mutant and WT plants, respectively. On average, 92.04% of Li_1_ mutant reads and 92.48% of WT reads were successfully mapped to the newly released *G*. *hirsutum* (TM-1) whole genome sequence [30]. Of the mapped reads, between 67.89% and 81.69% could be uniquely mapped to the TM-1 genome (S1 and S2 Tables).

### Comparison of DEGs between Li_1_ mutant and wild type during early fiber development stages

**Fig 3:**
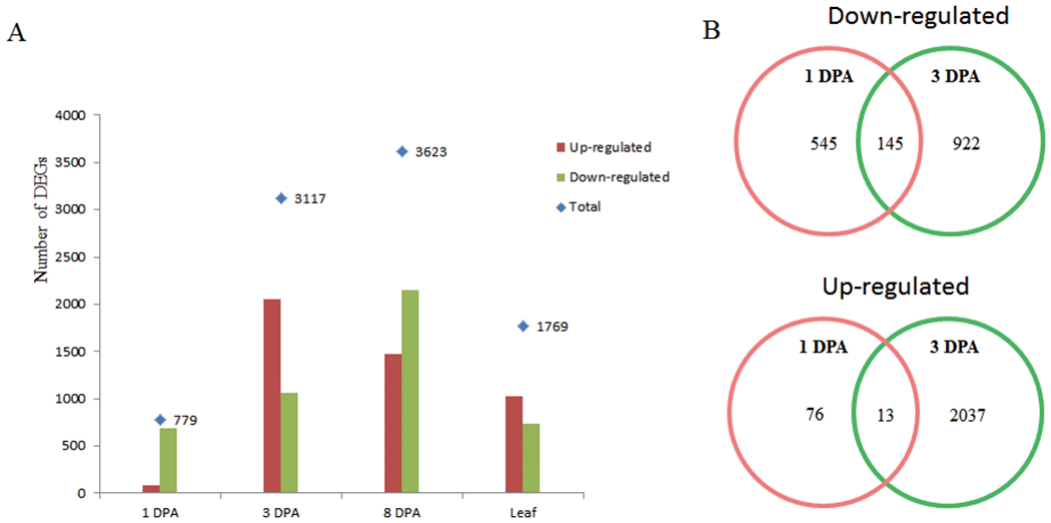
DEGs in the ovules and leaves of Li_1_ mutant and wild type plants. A: the expression levels of DEGs were altered in Li_1_ mutant compared to wild type; B: distribution of the DEGs at 1 DPA and 3 DPA.

All mapped sequenced reads for all identified transcripts were used for differential expression analysis in Cuffdiff, and those with a q value ≤ 0.05 and a fold-change between the Li_1_ mutant and WT of ≥ 2 were considered differentially expressed transcripts. Analysis of the data indicated that many genes showed differential expression in ovules and leaves. When transcriptomes from Li_1_ mutant and WT were compared, about 700–4,000 DEGs were identified in fibers from 1 DPA to 8 DPA and leaves. Only 779 genes were differentially expressed at 1 DPA, but 3117 genes were up-regulated or down-regulated at 3 DPA in Li_1_ mutant (Fig 3A).

Our SEM analyses revealed that Li_1_ mutant fibers become aberrant at 3 DPA, suggesting that altered gene expression may exist at or before this stage. This agrees with the results of previous studies [19]. Thus, comparing the gene expression profiles of 1 DPA and 3 DPA ovule tissues and analyzing common DEGs may enable us to gain an understanding of the regulation of gene expression in Li_1_ mutant fibers. When DEGs were compared across the two stages of fiber development, 145 DEGs were found to be consistently down-regulated and only 13 DEGs consistently up-regulated in 1 DPA and 3 DPA ovule tissues from Li_1_ mutant. Based on our annotation of TM-1 transcripts [30], we found that AUX/IAA transcriptional regulator and auxin-responsive related genes (8 genes), ethylene response transcription factors (16 genes), calcium transport and binding protein genes (8 genes), cell wall related genes such as xyloglucan endotransglucosylase/hydrolase family protein (XTH) and xyloglucan endotransglycosylase (XET) genes (3 genes), vesicle transport genes (3 genes), and zinc finger protein genes (11 genes) were down-regulated in Li_1_ mutant (S3 Table). We also found that two 1-aminocyclopropane-1-carboxylate oxidase (ACO) genes, two alcohol dehydrogenase 1 genes, and two heat shock protein genes were up-regulated in Li_1_ mutant. These results suggest that auxin, ethylene response, calcium-transport, and cell wall metabolism may play crucial roles in fiber early elongation (Fig 3B and S3 Table).

We also compared the DEGs at 1 DPA, 3 DPA, and 8 DPA, and found that 12 genes were consistently up-regulated (2 genes) or down-regulated (10 genes) in Li_1_ mutant (S4 Table). The two up-regulated genes were annotated as succinate dehydrogenase and a protein kinase superfamily protein. One down-regulated gene was annotated as a zinc finger family protein, and two as exocyst subunit exo70 family protein H7. In addition, 40, 1884, and 1287 up-regulated DEGs were only found at 1 DPA, 3 DPA, and 8 DPA, respectively and 489, 771, and 1928 down-regulated DEGs were found only at 1 DPA, 3 DPA, and 8 DPA, respectively (S2 Fig).

### Gene function annotation by GO enrichment analysis

**Fig 4:**
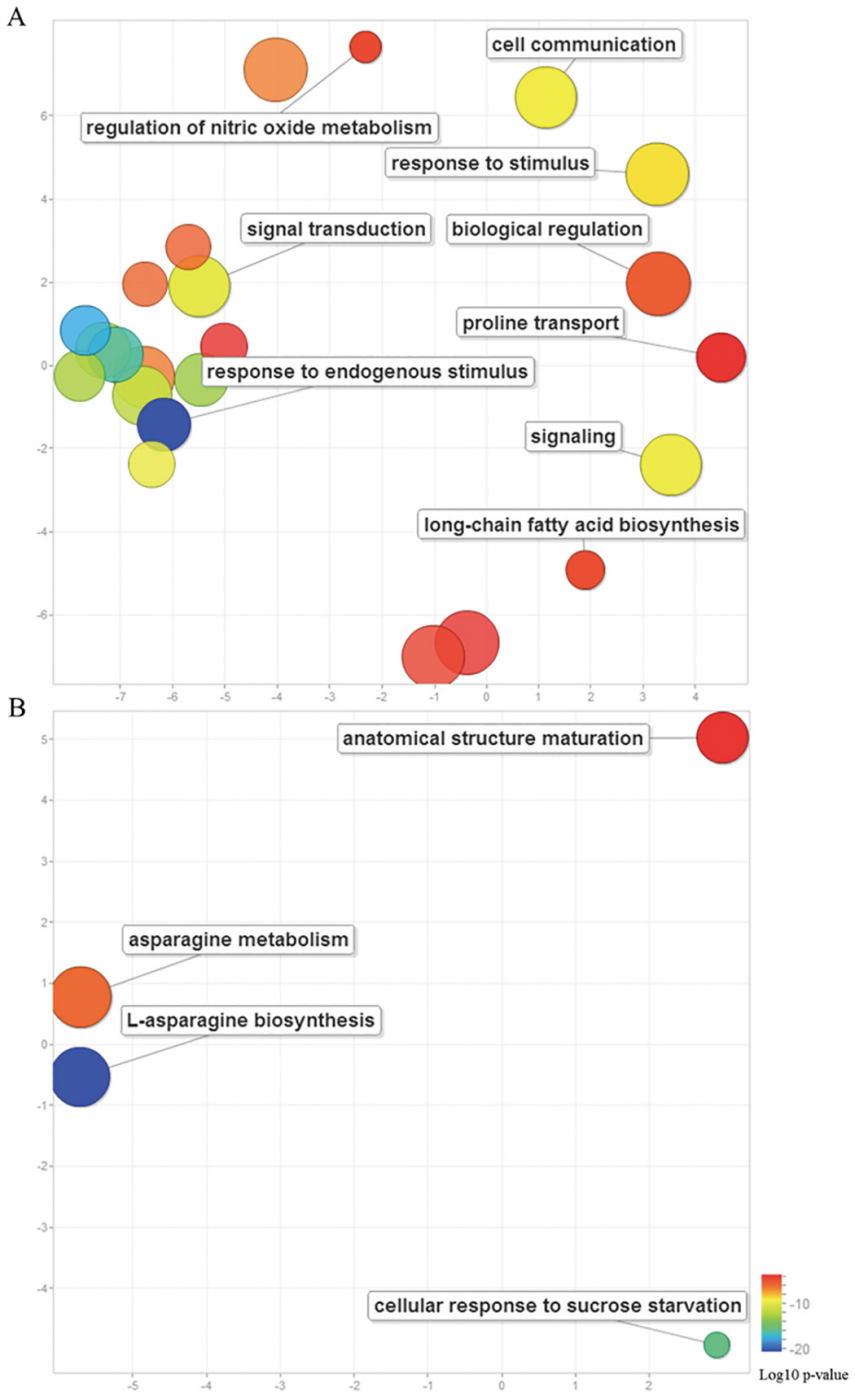
GO enrichment of common DEGs at 1 DPA and 3 DPA in Li_1_ mutant and WT tissues. Scatterplots of enriched GO terms for common down-regulated DEGs (A) and common up-regulated DEGs (B).

To understand the possible biological functions in fiber morphology that are modulated by the *Li*_*1*_ gene, GO functional enrichment was performed using an FDR adjusted p-value of <0.05 as the cutoff for the common DEGs at 1 DPA and 3 DPA. For a full list of GO terms see S5 Table. To understand the hierarchy of GO systems, the ReviGO tool was used to collectively visualize the enriched GO terms for DEGs [39]. Comparisons of these GO term lists revealed 145 down-regulated DEGs common to 1 DPA and 3 DPA tissues. The GO term responses to endogenous stimuli (GO:0009719), including response to ethylene, response to oxygen-containing compounds, and response to lipids, were enriched. The GO terms signal transduction, response to stimulus, cell communication, biological regulation, and long-chain fatty acid biosynthetic process were also identified. In addition, the enriched GO terms related to molecular functions were the xyloglucosyl transferase pathway, which is known to be involved in fiber elongation [41], polygalacturonate 4-alpha-galacturonosyltransferase activity, and sphingosine N-acyltransferase activity (GO:0050291), indicating that cell wall synthesis is compromised in Li_1_ mutants (Fig 4A and S5 Table). The 13 up-regulated DEGs were all found to be enriched in the biological processes of asparagine metabolism, L-asparagine biosynthesis, and the cellular response to sucrose starvation (GO:0043617) (Fig 4B and S5 Table). These results indicate the powerful regulatory functions of the *Li*_*1*_ gene as well as the complexity of the fiber elongation process.

### Comparison of DEGs in ovule and leaf tissues in Li_1_ mutant

**Fig 5:**
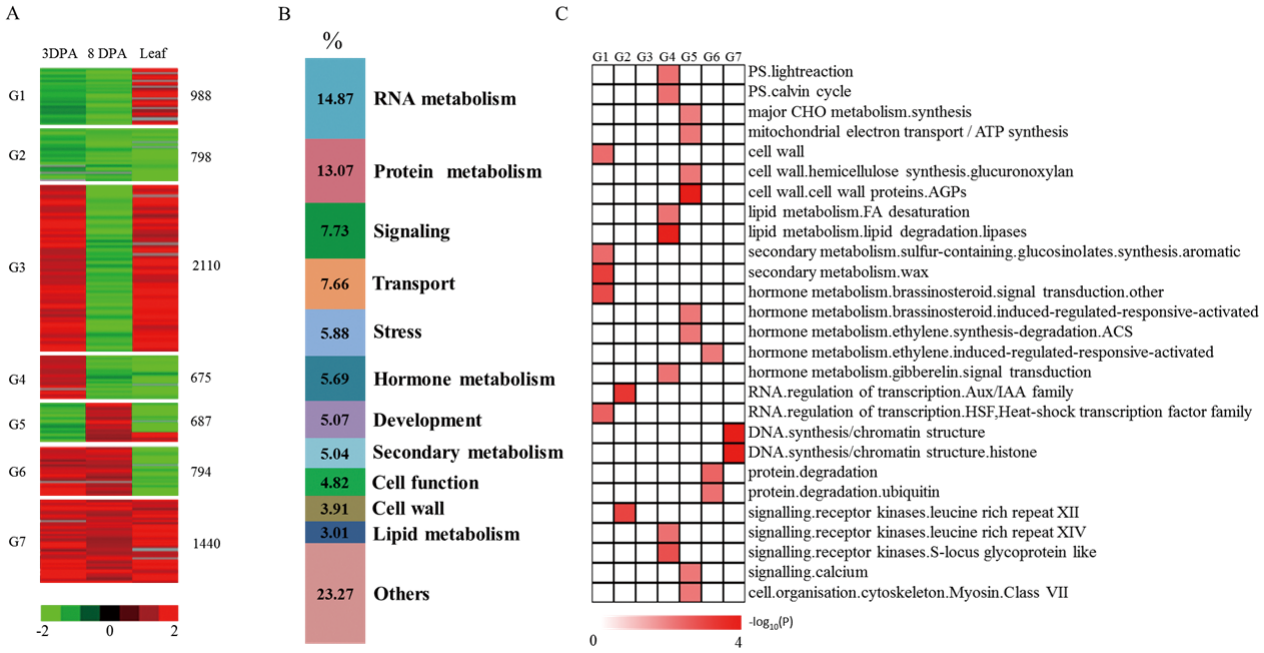
Dynamic progression of common DEGs in leaf and ovule transcriptomes. (A) Unsupervised hierarchical clustering of the 7492 common DEGs in the Li_1_ mutant. Common DEGs were clustered into six groups and the number of genes in each group is listed on the right. Red region, genes up-regulated in the Li_1_ mutant; green region, genes down-regulated in the Li_1_ mutant. (B) Functional distribution of common DEGs in the Li_1_ mutant. (C) Functional categorization of common DEGs in the dominant mutants. ‘Others’ includes 22 minor categories.

The phenotype of the Li_1_ mutant involves both vegetative tissues and fiber tissues. Thus, comparing the transcriptomes of leaf and ovule tissues and analyzing DEGs may help to uncover shared molecular mechanisms in fiber development and leaf morphogenesis. In our analysis, we compared the stages of fiber and leaf development in which the phenotype was altered (3 DPA and 8 DPA) and identified a total of 7492 DEGs with the potential to affect the development of both tissues. These DEGs were classified into seven groups (G1- G7) according to their expression patterns (Fig 5A). Of these, 988 genes in G1 were down-regulated at 3 DPA and 8 DPA ovules and were up-regulated in leaves; 798 genes in G2 were down-regulated at 3 DPA and 8 DPA ovules and in leaves; 2110 genes in G3 were highly expressed at 3 DPA ovules and in leaves; 675 genes in G4 were highly expressed at 3 DPA ovules; 687 genes in G5 were highly expressed at 8 DPA ovules; 794 genes in G6 were highly expressed at 3 DPA and 8 DPA ovules, and 1440 genes in G7 were highly expressed at 3 DPA and 8 DPA ovules and in leaves in Li_1_ mutants.

To identify biological pathways that may be altered in the Li_1_ mutant ovules and leaves, an enrichment test was carried out using a MapMan functional category, which annotated 5953 genes, excluding 23.27% belonging to the ‘not assigned’ or ‘unknown’ categories. Among these genes, 14.87% were related to RNA metabolism, 13.07% were related to protein metabolism, 7.73% were related to signaling, 7.66% were related to transport, and the remaining genes were related to processes such as stress, hormone metabolism, development, secondary metabolism, cell function, cell wall and lipid metabolism (Fig 5B). We found that light-reaction-, Calvin cycle-, and major CHO metabolism-related genes were mainly in G4 and G5. DEGs involved in cell wall metabolism, secondary metabolism, and the heat-shock transcription factor family, which was down-regulated in ovule tissues but up-regulated in leaves, were found in G1. However, ethylene metabolism- and protein degradation-related genes were up-regulated in ovules and down-regulated in leaf tissues. Plant hormones play an important regulatory role in plant growth and development. In this study, hormone metabolism-related genes, such as brassinosteroid and ethylene signal transduction genes were mainly found in G5. Interestingly, the DEGs in G2 were all down-regulated at 1 DPA, 3 DPA, and 8 DPA ovules and in leaves that were also enriched in auxin and receptor kinases. The expression pattern of genes in G7 was the opposite of that in G2, and chromatin structure-related genes were enriched in this group (Fig 5C). To better visualize the connections between leaf morphogenesis and fiber early elongation in Li_1_ mutant, we mapped 7492 DEGs in leaves and ovules in the KEGG database. In total, we assigned 1582 genes to KEGG pathways; most of which were related to sugar, secondary metabolite and lipid metabolism pathways; for example, 110 genes were annotated as being involved in starch and sucrose metabolism, 47 genes in pentose and glucuronate interconversions, 42 genes in flavonoid biosynthesis, and 39 genes in fatty acid degradation. These results suggest that the regulation of some enzymes that catalyze sucrose, starch, and glucose metabolism may directly or indirectly impact on fiber and leaf development (S6 Table).

### The role of very-long-chain fatty acid biosynthesis, hormone metabolism, cell wall development, and cytoskeleton development in fiber elongation

Fiber development-related genes, such as those involved in very-long-chain fatty acid (VLCFA) metabolic processes, show altered expression patterns in Li_1_ fibers. The biosynthesis of VLCFAs, which has important roles in plant growth and development, was found to significantly promote cotton fiber cell elongation in ovule culture medium [42]. Our data showed that most of the genes that were related to VLCFA synthesis were down-regulated in Li_1_ mutant, and only a few genes were up-regulated. Interestingly, we noted that 37 genes involved in the VLCFA metabolism process were suppressed in Li_1_ mutant during fiber development. Most of these were 3-ketoacyl-CoA synthase (KCS) genes (31 genes), an enzyme known to be rate-limiting in VLCFA biosynthesis [43, 44]. The other genes were ten AMP-dependent synthetase and ligase family protein (LACS) genes, one 3-oxo-5-alpha-steroid 4-dehydrogenase family protein (ECR) gene, and two protein-tyrosine phosphatase-like (HCD) genes. Notably, only a few genes showed differential expression at 1 DPA; most were differentially expressed at 3 DPA and 8 DPA, suggesting that VLCFA metabolism-related genes play important roles in early fiber elongation. However, in leaf tissues, most of these genes were up-regulated in Li_1_ mutant, with only a small number down-regulated. It is possible that these genes have different functions in leaf development (S7 Table).

It has been reported that plant hormones are considered to play important roles in regulating fiber development. A large number of genes involved in the metabolism of auxin, ethylene, abscisic acid (ABA), brassinosteroid (BR), cytokinin, and gibberellin were down-regulated in Li_1_ mutant. The expression of genes involved in ethylene and auxin synthase and response related genes were more dramatically altered than that of other hormones. Fifty-nine genes related to ethylene response and synthesis were significantly differentially expressed, with the majority being down-regulated in Li_1_ mutant. Four genes were annotated as being related to 1-aminocyclopropane-1-carboxylic acid synthase (ACS); the key enzyme in ethylene biosynthesis. These genes were down-regulated during early fiber elongation. Most ethylene-forming enzyme (ACO) genes, another key enzyme in ethylene biosynthesis, were down-regulated at 1 DPA, but up-regulated at 3 DPA in Li_1_ mutant. Furthermore, ethylene response factor (ERF) type genes (8 genes) and ethylene responsive element binding factor genes (12 genes) were down-regulated in early fiber elongation; especially at 1 DPA. Fifty auxin-responsive protein genes, including auxin-responsive GH3 family protein genes and the SAUR-like auxin-responsive protein family gene, demonstrated significant down-regulation at 1 DPA, 3 DPA, and 8 DPA in Li_1_ mutant. Moreover, three genes involved in brassinosteroid-6-oxidase were also down-regulated at 3 DPA and 8 DPA, and only one of them was up-regulated in leaves. Cytokinin oxidase and cytokinin oxidase/dehydrogenase genes were mainly down-regulated during fiber elongation and leaf development, except at 3 DPA, and most gibberellin 20 oxidase and gibberellin 2-oxidase genes were down-regulated at 3 DPA and 8 DPA (S8 Table).

A number of cell wall biosynthesis genes were differentially expressed during fiber elongation in the Li_1_ mutant compared to the WT; most of which showed significant differences in expression at 3 DPA and 8 DPA, but not at 1 DPA. These results indicate that changes in the expression of cell wall-related genes occurred after the fiber developmental anomaly, and the altered expression patterns of these genes may be due to upstream genetic regulation (S9 Table). Expansin is an important cell wall structural protein. In this study we found that expansin and expansin-like genes were down-regulated in Li_1_ mutant and that more of these genes were significantly differentially expressed at 3 DPA and 8 DPA. Several genes related to XTH, arabinogalactan, glycosyl hydrolase, pectin lyase-like, and UDP-D-glucose metabolism were also down-regulated at 3 DPA and 8 DPA, and three XETs and four XTHs family protein genes were mainly down-regulated at 1 DPA and 3 DPA. In addition, our study revealed that numerous genes related to the cytoskeleton, including actin, tubulin, and myosin, exhibited changes in expression in Li_1_ mutant compared to WT. Most actin genes (11 genes) and tubulin genes (25 genes), including isoforms of tubulin alpha-3, tubulin alpha-2 chain, beta-6 tubulin, tubulin beta 8, and tubulin beta chain 3, were down-regulated in Li_1_ mutant, especially at 8 DPA. Many other DEGs related to microtubule associated protein, myosin family protein, and villin were down-regulated at 8 DPA, but most were up-regulated in leaves (S10 Table).

These results indicate that numerous genes expressed during fiber development are regulated by the *Li*_*1*_ gene as early as the fiber initial stage, and the subsequent reprogrammed gene expression at 3 DPA, suggesting that the molecular mechanisms regulated by the mutant gene may play crucial roles in fiber elongation. This suggests that 3 DPA is likely to be the point where fiber elongation is altered in Li_1_ mutant.

### Validation of differentially expressed genes by qPCR

**Fig 6:**
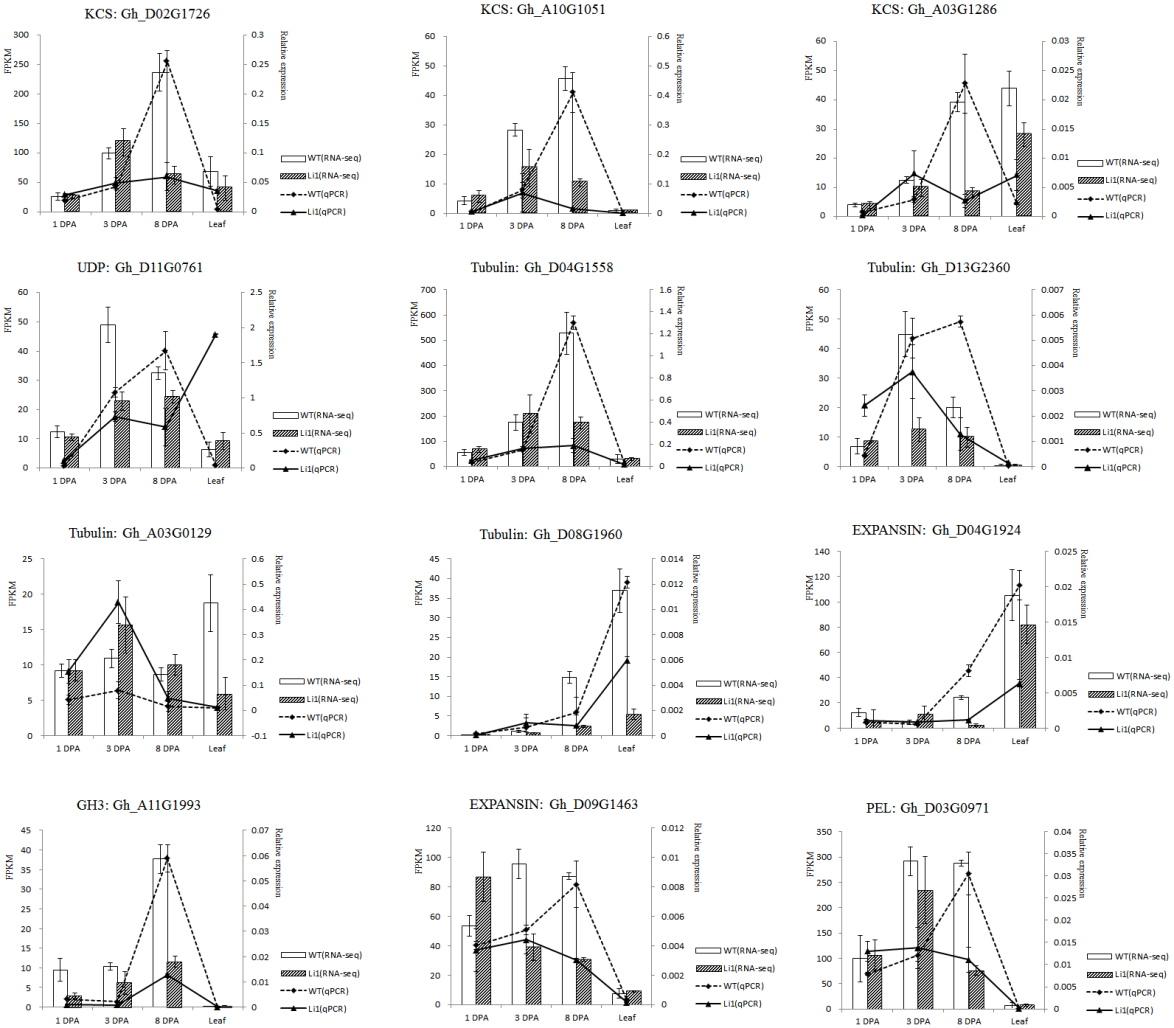
RNA-seq and qPCR analyses of transcription levels of fiber development related genes in Li_1_ mutant and WT plants. **A-I: qPCR analysis of gene expression levels** in 1 DPA, 3 DPA, and 8 DPA ovules and in leaves; J-R: Transcriptome expression levels of these genes.

To determine whether the RNA-seq gene expression results were reliable, we also used RNA samples isolated for RNA sequencing to perform qPCR analysis. The genes selected for RNA-seq corroboration by qPCR were chosen primarily from DEGs between Li_1_ mutant and WT leaf and ovule tissues. qPCR showed similar expression profiles to those from the RNA-seq analysis (Fig 6 and S11 Table).

## Discussion

### The Li_1_ short fiber trait is caused by gene regulation at early stages of fiber development

Cotton fibers are highly elongated single-celled seed trichomes that emerge from ovule epidermal cells on or about DOA [1]. Fibers can grow to 30 to 40 mm in length [6, 45–47]. Li_1_ is a naturally occurring mutant in upland cotton (*Gossypium hirsutum*) with the phenotype of extremely short fibers and distorted leaves. This makes it a very useful material for studying the molecular events controlling fiber and leaf development [19]. Earlier SEM studies indicated that Li_1_ mutant can initiate fibers normally from ovule epidermal cells at anthesis, and the fibers’ morphologies are similar to WT before 3 DPA, when aberrant fiber morphology occurs [19]. Previous studies of fiber development in the Li_1_ mutant have focused on SCW synthesis and reports of altered molecular events at early stages of fiber development are limited [21, 24]. Proteomic analysis of 12 DPA Li_1_ mutant fibers demonstrated significant perturbation of expression profiles [27]. Recent microarray profiling suggested that 6 DPA is likely to be a critical period of early fiber elongation because many genes displayed significantly altered expression at this point compared to 0 DPA and 3 DPA [26]. Therefore, the stage of fiber elongation reaches a critical turning point in Li_1_ mutant remains uncertain. In our study, SEM analyses revealed that Li_1_ mutant fiber morphology was abnormal at 3 DPA compared to WT fibers, and this was supported by the results of earlier SEM studies. In addition, our transcriptome sequencing showed that the number of DEGs at 3 DPA was four times that at 1 DPA (779 vs. 3117). According to these results, 3 DPA is likely to be a critical point in early fiber elongation in Li_1_ mutant (Figs 2 and 3; S1 Fig).

GO analysis of common DEGs at 1 DPA and 3 DPA showed that genes involved in responses to stimuli and signal transduction were significantly enriched in Li_1_ mutant (Fig 4). Our data show that 11 ethylene-responsive transcription factors, 7 auxin-induced proteins, and calcium-binding/transporting proteins were down-regulated in Li_1_ mutant, demonstrating that ethylene, auxin, and calcium play an important role in the early stages of fiber elongation. Moreover, phosphate-responsive 1 family protein (7 genes), and zinc finger protein (11 genes) were also down-regulated in Li_1_ mutant (S3 Table). Our results support the speculation that ethylene and auxin play a positive role in cotton fiber elongation [42, 48].

### Ethylene and VLCFA metabolism play an important role in fiber elongation in Li_1_ mutant

**Fig 7:**
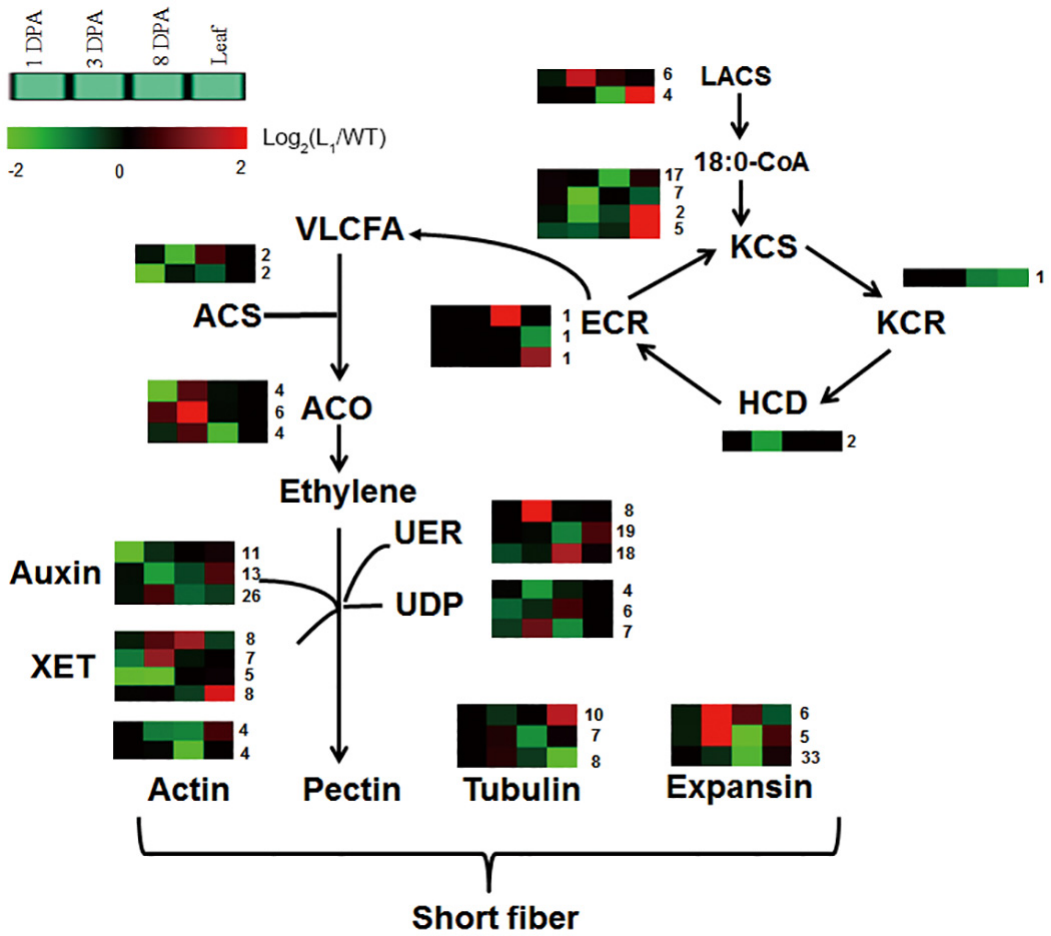
Early fiber elongation pathways that are differentially regulated in Li_1_ mutant and WT plants.

VLCFAs are not only an important biological membrane component, but also play a key role in cell growth and development, and during the early stages of fiber elongation development, the genes related to VLCFAs biosynthesis were found to be significantly altered. It is speculated that free fatty acids or their derivates may serve directly as signaling molecules in plants: VLCFAs promote cotton fiber cell elongation exogenously in ovule culture medium, and *Gh*KCSs codes for 3-ketoacyl-CoA synthase (KCS), which is a key enzyme in the VLCFA synthesis process [42]. Our data demonstrated that dozens of genes related to VLCFA synthesis, including 31 KCS genes, were repressed in Li_1_ mutant; suggesting that they are required for normal fiber cell expansion (S7 Table, Fig 7).

Ethylene plays a positive role in cotton fiber cell elongation at early stages of fiber elongation. It was reported previously that there is increased ACS activity during cotton fiber elongation, and microarray profiling has revealed that some ACS and ACO genes are significantly down-regulated in Li_1_ mutant [21, 26, 49]. In our study, two ACS genes (Gh_A12G2673, Gh_D12G2746) were down-regulated more than 5-fold at 1 DPA, and another two ACS genes (Gh_A02G1046, Gh_D03G0678) demonstrated more than 3-fold down-regulation at 3 DPA. Moreover, we found that many ERF domain proteins and ethylene responsive element binding factors were down-regulated at 1 DPA and 3 DPA. The expression pattern of ethylene-forming enzyme (ACO) was similar to that of ethylene responsive element binding factor, suggesting that they may be involved in early fiber elongation stages (S8 Table, Fig 7).

### Specific proteins involved in fiber elongation and leaf morphogenesis in Li_1_ mutants

The mutant phenotype of Li_1_ involves both fiber and vegetative tissues such as leaves, stems, and flowers. Microarray and proteome profiling technologies have been used to study DEGs in Li_1_ mutant fibers [21, 26, 29]. However, few studies have investigated the process of DEGs in Li_1_ mutant leaf tissues [28]. Studying the pathways of key regulators could contribute to revealing the reason for the termination of fiber elongation and leaf morphology abnormalities in Li_1_ mutant. In this study we used NGS technology to profile changes in ovule and leaf tissues from the Li_1_ mutant and WT, since it is likely that these two tissues share common developmental pathways. This will allow a clearer understanding of the mechanisms of early fiber elongation and leaf morphogenesis regulation in Li_1_ mutant.

From 1 DPA to 8 DPA, fiber cells are differentiated from the ovules surface and elongate rapidly. Our data showed that DEGs in leaf tissues and ovule tissues involved in RNA metabolism, protein metabolism, and signaling categories were significantly enriched in mutant plants. A large number of RNA metabolism-related genes were detected along with a dramatic increase in the total number of ribosomes observed in the zygote [50]. Transcription factors play an important role in the regulation of gene expression. In the present study, zinc finger family, AP2/B3, ERF, MYB transcription factor family, and bZIP transcription factor family genes were readjusted by the Li_1_ mutation in 1 DPA, 3 DPA, and 8 DPA ovules (S12 Table).

Genes that have been reported to be significantly overexpressed during fiber initiation and elongation can be divided into several functional subgroups: those related to cell wall metabolism, reactive oxygen species (ROS) homeostasis, cytoskeleton arrangement, hormone signal transduction, and transcription factors [51–54]. In this study, we found that gene groups that function in elongation are also differentially expressed in Li_1_ mutant leaf tissues and ovules, indicating shared regulatory pathways in the two processes.

Tubulins are important cytoskeleton proteins and play key roles in fiber cell elongation [55–58]. Overexpression of *β*-tubulin-like cDNA induced longitudinal growth in yeast cells [58]. Therefore cell morphology is affected by cytoskeletal proteins. The *γ*-tubulin gene has been found to be differentially expressed: It was down-regulated by more than 2-fold in 24 DPA fibers in the Li_1_ mutant [24]. In our study, cytoskeleton-related genes showed no difference in expression in 1 DPA ovules, but were down-regulated in 3 DPA and 8 DPA ovules, and up-regulated in leaf tissues. Such variations may be due to the fact that the cytoskeleton proteins play different regulatory roles in different tissues (S10 Table).

The plant cell wall is a complex structure whose functional integrity is constantly being monitored and maintained during development, and the integrity of the cell wall structure is an important component of many metabolic processes [59–60]. Previous studies have shown that plants that overexpress GhXTH1 have increased XTH activity and produce mature cotton fibers that are 15% to 20% longer than those of WT cotton plants under both greenhouse and field growth conditions [41]. In the current study, several cell wall metabolism genes, including EXPANSIN, Glycosyl hydrolase, FASCICLIN-like, and XTH genes were down-regulated during fiber elongation in Li_1_ mutant a result that is similar to those in previous reports. In particular, several EXPANSINS, rhamnogalacturonate lyase family proteins, and XTHs were up-regulated in leaf tissues. These results suggest that cell wall metabolism-related proteins play important roles in leaf morphology and fiber elongation (S9 Table).

### Functional analysis of DEGs on chromosome 22

Li_1_ is a monogenic dominant mutant, and using SSR mapping the causative locus has been assigned to chromosome 22. Recent results show that the marker TMB2500 is 0.8 cM away from the *Li*_*1*_ locus [19–21]. Moreover, previous results suggest that the *Li*_*1*_ locus might be close to the centromeric region [20]. Since the allotetraploid cotton genome is rich in repeat sequences, the fine mapping of mutated genes through the molecular markers alone is difficult. Using the genome sequence of TM-1 [30] and transcriptome analysis, the identification DEGs throughout chromosome 22 can help us to effectively narrow the range of candidate genes. Our data show that there are 219 genes that are differentially expressed in chromosome 22. Among these genes, fiber development-related genes, such as peroxidase superfamily protein, UDP-Glycosyltransferase superfamily protein, EXPANSIN, and myb domain protein genes, were significantly up- or down-regulated at 3 DPA and 8 DPA. Of the 19 DEGs identified at 1 DPA, 15 were down-regulated. One gene was annotated as the galacturonosyl transferase-like 10 gene, which was down-regulated at 1 DPA and 3 DPA and has been located to position 8.13–8.14 Mb of chromosome 22 (S13 Table).

In conclusion, although the short fiber phenotype of Li_1_ may be determined by a single gene, transcriptome comparison analysis of the Li_1_ mutant and WT indicates that the down-regulation of several important fiber development-related pathways may be the major reason for such phenotypes. In this study, we first used the tetraploid genome sequence as a reference for transcriptome analysis. We then compared leaf and ovule tissues from mutants and WT during primary fiber elongation and leaf development. Critical genes and pathways involved in VLCFA synthesis, cell wall metabolism, the cytoskeleton, and hormone metabolism may play major roles in the pleiotropic phenotype of the Li_1_ mutant. Our study of the Li_1_ mutant provides additional detail and new insights into the mechanisms of early fiber elongation.

## Supporting Information

S1 Fig SEM analysis 1 DPA and 3 DPA of WT (A,C) and Li1 mutant (B, D) fibers. A,B: 1 DPA; C,D: 3 DPA; bar = 50μm.
(TIF)

S2 Fig Statistical of DEGs between Li_1_ and WT at 1 DPA, 3 DPA and 8 DPA. A: Up-regulated DEGs in Li_1_ mutant, B: Down-regulated DEGs in Li_1_ mutant.
(TIF)

S1 Table Statistics of transcriptome sequencing for the 24 transcriptome libraries. (XLSX)

S2 Table Percentage of short reads mapped to genome in Li_1_ mutant and WT. (XLSX)

S3 Table List of 158 common DEGs at 1 DPA and 3 DPA between the Li_1_ mutant and WT. (XLSX)

S4 Table List of 12 common DEGs at 1 DPA, 3 DPA and 8 DPA between the Li_1_ mutant and WT. (XLSX)

S5 Table GO enrichment analysis of common DEGs at 1 DPA and 3 DPA. (XLSX)

S6 Table KEGG analysis of DEGs in ovules and leaves. (XLSX)

S7 Table Putative very-long-chain fatty acid metabolism gene between Li_1_ and WT. (XLSX)

S8 Table Putative hormone related DEGs between Li_1_ and WT. (XLSX)

S9 Table Putative cell wall related DEGs between Li_1_ and WT. (XLSX)

S10 Table Putative cytoskeleton-related DEGs between Li_1_ and WT. (XLSX)

S11 Table qPCR primers for twelve genes. (XLSX)

S12 Table Differentially expressed transcription factors in Li_1_ mutant. (XLSX)

S13 Table List of 219 DEGs at chromosome 22. (XLSX)
