# Supplementary figures and images for: Transcriptome Analysis of Short Fiber Mutant Ligon lintless-1 (Li1) Reveals Critical Genes and Key Pathways in Cotton Fiber Elongation and Leaf Development
Source: PLoS One. 2015 Nov 24;10(11):e0143503. doi: 10.1371/journal.pone.0143503 (PMC4658197; doi:10.1371/journal.pone.0143503)

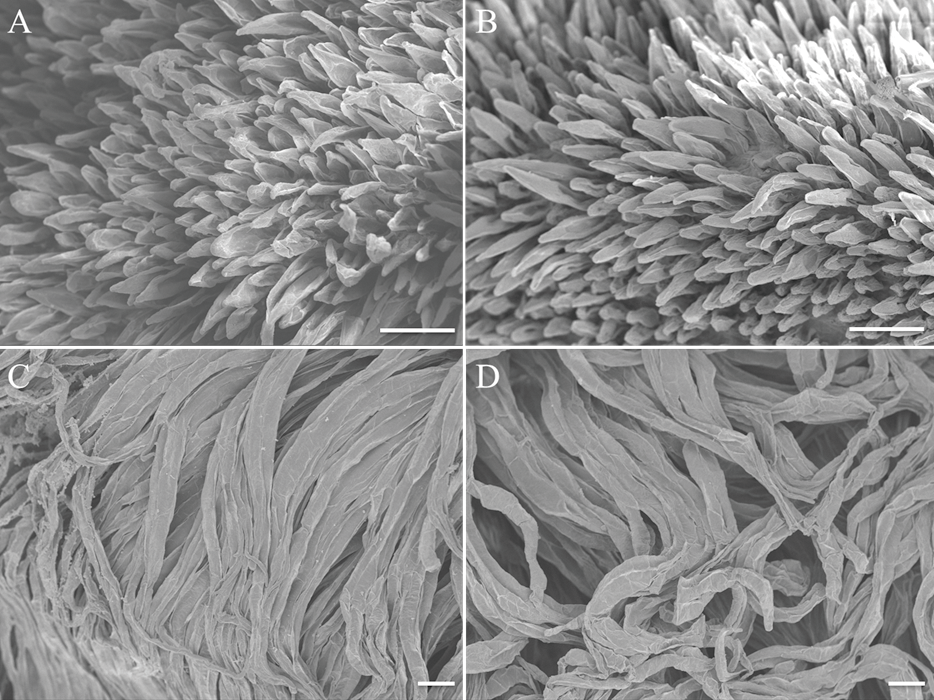

Supplement: S1 Fig — A,B: 1 DPA; C,D: 3 DPA; bar = 50μm. (TIF) [file pone.0143503.s001.tif]

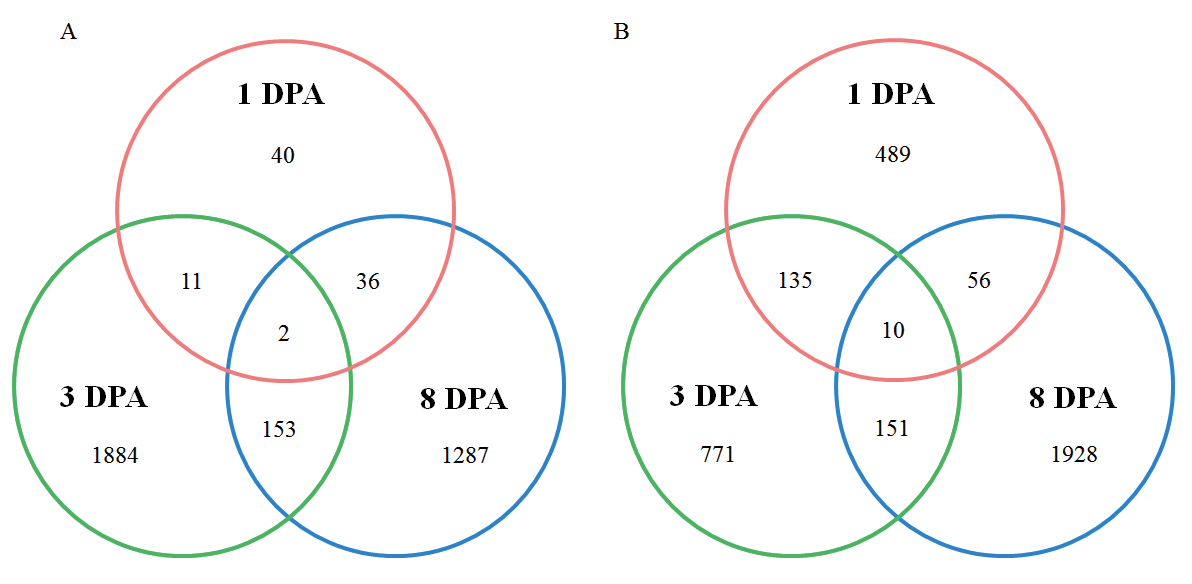

Supplement: S2 Fig — A: Up-regulated DEGs in Li1 mutant, B: Down-regulated DEGs in Li1 mutant. (TIF) [file pone.0143503.s002.tif]
